# Gate Wait for Better Air

**DOI:** 10.1289/ehp.119-a288a

**Published:** 2011-07-01

**Authors:** Carol Potera

**Affiliations:** Carol Potera, based in Montana, has written for *EHP* since 1996. She also writes for *Microbe*, *Genetic Engineering News*, and the *American Journal of Nursing*.

Commercial jets may spend as much as 30% of their total flight time taxiing on the tarmac before takeoff.^1^ In the United States, this “taxi-out time” translates into 6 million metric tons of carbon dioxide, 45,000 metric tons of carbon monoxide, 8,000 metric tons of nitrogen oxides, and 4,000 metric tons of hydrocarbons emitted into the atmosphere yearly.^1^ Holding planes at the gate for fewer than 5 extra minutes may be a simple way to reduce these emissions, according to a pilot study conducted at Boston Logan International Airport by researchers at the Massachusetts Institute of Technology (MIT).^2^

The MIT team studied departure data from Logan and developed models to minimize runway congestion by increasing the amount of time planes spent at the gate. Working with the Federal Aviation Administration (FAA) and air traffic controllers, the MIT team ran eight 4-hour tests in August and September 2010 during Logan’s busiest arrival and departure times.

The average taxi-out time at Logan is about 20 minutes. Holding 247 flights at the gate for an average of 4.3 extra minutes reduced taxiing time by an average of 20%, and fuel consumption dropped by 16–20 gallons per plane. Takeoff times were not delayed, because once planes pushed back they proceeded quickly to takeoff. About 18 hours of taxi-out time were eliminated over the course of the study, resulting in an overall estimated fuel savings of 3,900–4,900 gallons.

This simple strategy offers a potential win–win situation for airports, airlines, and neighboring communities whose air is polluted by airport emissions. Air traffic controllers and aircraft pilots “were very positive and liked the fluid flow of aircraft on the ground instead of long queues,” says study leader Hamsa Balakrishnan. She’s working with the FAA to improve the method and test it at other congested airports. The team is also working on a model to estimate emissions avoided through the reduction in fuel consumption.

Hazardous air pollutants measured at or near airports include nitrogen dioxide, polycyclic aromatic hydrocarbons, fine particles, carbonyls, and volatile organic compounds.^3^^,^^4^^,^^5^ These pollutants have been generally linked to cancer,^6^ heart attack,^7^ and type 1 diabetes.^8^ In one study, people living within 5 miles of airports were 1.5 times more likely than people living farther away to be admitted to hospitals for a variety of respiratory diseases.^9^

“It’s important that we minimize emissions,” says Lourdes Maurice, executive director for environment and energy at the FAA. Although it remains to be seen whether reduced taxi-out times at other airports will translate into significant fuel savings and emissions reductions, Maurice says, “The initial benefits look very good and we are continuing efforts with MIT.”
